# Transcriptome Analysis Reveals Differential Expression of Genes Regulating Hepatic Triglyceride Metabolism in Pekin Ducks During Dietary Threonine Deficiency

**DOI:** 10.3389/fgene.2019.00710

**Published:** 2019-08-02

**Authors:** Yong Jiang, Ming Xie, Wenlei Fan, Jiajia Xue, Zhengkui Zhou, Jing Tang, Guohong Chen, Shuisheng Hou

**Affiliations:** ^1^Key Laboratory of Animal (Poultry) Genetics Breeding and Reproduction, Ministry of Agriculture and Rural Affairs; Institute of Animal Sciences, Chinese Academy of Agricultural Sciences, Beijing, China; ^2^College of Animal Science and Technology, Yangzhou University, Yangzhou, China; ^3^College of Food Science and Engineering, Qingdao Agricultural University, Qingdao, China

**Keywords:** threonine deficiency, triglyceride accumulation, fatty acid oxidation, energy metabolism, fatty acid profile, Pekin duck

## Abstract

Dietary threonine (Thr) deficiency increases hepatic triglyceride accumulation in Pekin ducks, which results in fatty liver disease and impairs hepatic function. However, the underlying molecular mechanisms altered by dietary Thr deficiency are still unknown. To identify the underlying molecular changes, 180 one-day-old ducklings were divided into three groups, including Thr deficiency group (Thr-D), Thr sufficiency group (Thr-S), and pair-fed group (Pair-F) that was fed with a Thr-sufficient diet but with reduced daily feed intake. The results showed that feed intake was similar between Thr-D and Pair-F groups, but weight gain rate and final body weight in the Thr-D group were lower than those in the Pair-F group. Feed intake, weight gain, and body weight in Thr-D and Pair-F groups were lower than those in the Thr-S group. The Thr-D diet reduced abdominal fat percentage but increased hepatic triglyceride content when compared with that of the Thr-S and Pair-F groups. The Pair-F reduced hepatic levels of C15:0, C17:0, C18:0, C20:0, C20:4n6, and C22:0 and also reduced total fatty acid, saturated fatty acid, and unsaturated fatty acid content when compared with those of the Thr-D and Thr-S groups. The Thr-D diet increased hepatic content of C6:0, C17:1, C18:3n6, C20:0, C20:1n9, and C22:2, as well as reduced the content of C18:2n6t and C23:0 when compared with those of the Thr-S group. Transcriptome analysis in the liver indicated that the Thr-D diet upregulated genes related to fatty acid and triglyceride synthesis and downregulated genes related to fatty acid oxidation and triglyceride transport. Gene ontology analysis showed that more genes related to lipid metabolism processes and molecular function were differentially expressed in the Thr-D group relative to Thr-S and Pair-F groups than in the Pair-F group relative to the Thr-S group. KEGG pathway analysis showed that differentially expressed genes were enriched in signal transduction, immune, hormone, lipid, and amino acid metabolism pathways. Our findings indicated that the Thr-D diet increased hepatic triglyceride and fatty acid accumulation *via* increasing fatty acid and triglyceride synthesis and reducing fatty acid oxidation and triglyceride transport. These findings provide novel insights into our understanding of the molecular mechanisms underlying fat accumulation in the liver caused by dietary threonine deficiency.

## Introduction

Nonalcoholic fatty liver disease (NAFLD) is an increasingly prevalent disease worldwide that leads to chronic hepatic injury (Loomba and Sanyal, 2013). It is estimated that NAFLD affects approximately 25% of the world population (Lazo and Clark, 2008; Rector et al., 2008). Recently, the occurrence of NAFLD has been increasing in adolescents as the prevalence of childhood obesity rises (Janssen et al., 2005; Park et al., 2005). Lipid accumulation in the liver of people who drink little or no alcohol is the main characteristic of NAFLD. Such a fatty liver is more susceptible to inflammatory cytokines and oxidative stress (Browning and Horton, 2004; Cohen et al., 2011) and may gradually develop into steatohepatitis, liver fibrosis, and hepatocellular carcinoma (Chalasani et al., 2012).

Threonine (Thr) is an essential amino acid for humans and poultry (Kidd and Kerr, 1996) and plays important roles in various metabolic processes, such as hormone secretion and immune defense. Its importance is highlighted by the adverse effects of Thr deficiency in experimental animals, including anorexia, impaired immunity, excessive energy expenditure, and growth retardation (Ross-Inta et al., 2009; Xie et al., 2014; Jiang et al., 2016; Zhang et al., 2016). For example, dietary Thr deficiency reduces the food intake of poultry (Xie et al., 2014; Zhang et al., 2014; Jiang et al., 2016; Zhang et al., 2017), whereas such a reduced feeding leads to low fat deposition in young growing cattle (Basarab et al., 2003). Moreover, a threonine-deficient diet elicits lipid accumulation in the liver of rats (Methfessel et al., 1964). In broilers, dietary Thr deficiency increases fat content in breast muscle and whole body (Rangel-Lugo et al., 1994; Ciftci and Ceylan, 2004). More recently, we demonstrated that dietary Thr deficiency increases hepatic lipid deposition and reduces abdominal fat levels in ducks (Jiang et al., 2017).

Hepatic lipid accumulation is a complex process, and the role of Thr in it remains elusive. It is speculated that Thr deficiency increases hepatic lipid deposition by changing lipid catabolic and transport pathways, as well as promoting lipid synthesis (Methfessel et al., 1964). Indeed, we have shown that the expression of several hepatic genes related to lipid uptake, fatty acid synthesis, β-oxidation, ketogenesis, and triglyceride transport is affected by dietary Thr levels in Pekin ducks (Jiang et al., 2019). Nevertheless, the biochemical mechanisms whereby Thr deficiency causes hepatic lipid accumulation in ducks are still unclear.

Therefore, the present study was conducted to investigate the effects of dietary Thr deficiency and feed restriction on the growth performance, plasma parameters, hepatic lipid content, and hepatic fatty acid composition. Moreover, we also assessed the expression of hepatic genes by transcriptome analysis to explore potential pathways whereby Thr deficiency causes triglyceride accumulation in the liver of Pekin ducks. Noteworthy, because the reduced food intake associated with Thr deficiency (Jiang et al., 2017) may affect hepatic lipid levels, it is necessary to compensate for this effect by designing a paired fed group. We used such a group in this study, whose feed consumption was similar between animals fed with Thr-sufficient and Thr-deficient diets.

## Materials and Methods

### Animals and Experimental Design

A completely randomized design with single factorial arrangement of treatments was used in this experiment. Dietary treatments included a Thr deficiency diet (Thr-D), a Thr sufficiency diet (Thr-S), supplemented with 0.21% (w/w) crystal Thr to basal diet as fed, and a paired fed group (Pair-F), which was fed with Thr sufficiency diet maintaining a similar daily feed intake to that of the Thr-D group by feed restriction. Thus, there were a total of three different treatments (Thr-D, Thr-S, and Pair-F).

One-day-old male Pekin ducks (Pekin Duck Breeding Centre of the Institute of Animal Science), 180 individuals, were randomly assigned to one of the three treatments composed of six replicate cages with 10 ducklings per cage according to average body weight (57.1 ± 3.01 g). All ducks were handled in accordance with the Pekin duck management guidelines. The birds were housed in raised wire floor pens (200 × 100 × 40 cm) with nipple drinkers and tubular feeders and maintained under constant light. The temperature was kept at 30°C from 1 to 3 days of age and then gradually reduced to 25°C until 21 days of age. Feed pellets and water were offered *ad libitum*.

The basal corn–wheat–peanut meal diet (**Supplementary Table S1**) was formulated to meet or exceed the current National Research Council recommendation (NRC, 1994), except for Thr concentration. First, a single batch of basal diet was mixed and then divided into two aliquots according to the experimental treatments. According to previous study (Zhang et al., 2014), each sublot was mixed with 0.21% crystalline Thr or cornstarch, which was used to replace crystalline Thr and maintain dietary characteristics. Dietary Thr concentrations in Thr-D and Thr-S diets were 0.53% (w/w) and 0.73% (w/w), respectively. Weight and feed intake per cage were measured weekly to calculate the daily body weight gain, daily feed intake, and gain:feed ratio from day 1 to day 21. All experimental procedures were approved by the Animal Management Committee (in charge of animal welfare issue) of the Institute of Animal Science, Chinese Academy of Agricultural Sciences (IAS20160322, IAS-CAAS, Beijing, China) and performed in accordance with the guidelines. Ethical approval on animal survival was given by the animal ethics committee of IAS-CAAS.

### Sample Collection and Preparation

At the 21^st^ day of age, ducks were fasted for 12 h and weighed individually to calculate the average body weight of animals in each cage for each treatment. Then, three birds whose weights were close to the average body weight of animals in each cage were selected. Blood samples were collected by jugular vein puncture with heparinized syringes equipped with stainless steel needles. The plasma was separated by centrifugation and stored at -20°C until analysis. Then, the selected birds were killed by cervical dislocation, and left liver samples were collected. A liver sample from the same location was frozen in liquid nitrogen for analysis of gene expression, and the left sample was frozen at -20°C for analyses of total lipids, cholesterol, total triglycerides, and fatty acid compositions. The three duck samples were pooled into one sample with same volume or weight in individual cage, and then, pooled samples were used for liver lipid analysis, fatty acid analysis, gene expression analysis. Other three birds were selected from each cage to collect breast muscle, thigh muscle, abdominal fat, and liver; these organs were weighed to calculate their weight in relation to the whole body. Finally, other three birds from each group were killed by cervical dislocation, and liver samples were collected and frozen in liquid nitrogen for transcriptome analysis.

### Dietary Protein and Amino Acids

Dietary protein levels were determined according to the Kjeldahl method (Thiex et al., 2002). The amino acid concentrations in diets were measured with an amino acid analyzer (L-800; Hitachi, Tokyo, Japan) after hydrolysis in 6-M HCl for 24 h at 110°C. Dietary tryptophan was determined according to the method of Official Journal of the European Communities (EU2000/45/EC).

### Plasma Parameters

The levels of plasma parameters, alanine transaminase (ALT), aspartate transaminase (AST), alkaline phosphatase (ALP), glucose, total triglycerides (TG), total cholesterol (CHO), high-density lipoprotein cholesterol (HDLC), and low-density lipoprotein cholesterol (LDLC), were measured using an automatic analyzer (Hitachi 7080, Tokyo, Japan) with commercial kit (Maccura, Sichuang, China).

### Liver Lipids

Total lipids were extracted by homogenizing minced liver tissue samples in chloroform–methanol (2:1, v/v) as described previously (Folch et al., 1957). The extracts were evaporated under a stream of nitrogen, weighed, and resuspended in chloroform–methanol (2:1) containing 0.01% (w/v) butylated hydroxytoluene. The concentrations of TG and CHO were measured using commercial kits (BioSino Bio-technology and Science Inc, Beijing, China).

### Fatty Acids Composition

Liver samples were prepared according to a previously described method (Yang et al., 2010a). Briefly, fatty acids were extracted from total lipids and methylated by adding 1-ml acetyl chloride/methanol (1:10, v/v) and 20 μl of 5 mg/ml nonadecanoic acid (used as internal standard) for 4 h at 80°C. Then 1 ml of hexane and 1.5 ml of 6% (w/v) K_2_CO_3_ were added into the tube. After shaking for 10 min, 400 μl of the hexane layer was transferred to a new injection vial after centrifugation for 10 min at 3,000×g for further gas chromatography–mass spectrometry (GC-MS) analysis. The fatty acid methyl esters were separated by GC (Agilent 6890, Agilent Technologies, Santa Clara, CA, USA) using a DB 23 capillary column (60 m × 0.25 mm × 0.25 μm, Agilent Technologies, Santa Clara, USA) with MS detection (5970C, Agilent Technologies, Santa Clara, CA, USA). Samples (1 μl) were injected using an autosampler. The oven was programmed as follows: 50°C for 1 min, ramp to 175°C at 25°C/min holding 3 min, ramp to 200°C at 3.5°C/min holding 3 min, and finally ramp to 230°C at 2°C/min holding 3 min with helium as the carrier gas at a split ratio of 1:50. The GC was operated at constant flow pressure of 33.357 kPa, and the injector temperature, transfer line, and ion source were 250, 250, and 230°C, respectively. Peaks were identified by comparing retention times with those of the corresponding standards (Sigma Aldrich, Saint Louis, MO, USA).

### RNA Isolation

Total RNA was extracted from frozen liver samples with RNAiso Plus reagent (code no. 9109, Takara, Dalian, China) according to the manufacturer’s instructions. For quantitative polymerase chain reaction (qPCR) analysis, RNA concentration was measured at 260 nm (NanoDrop™ 2000, Thermo Fisher Scientific, Waltham, MA, USA), and the RNA integrity was evaluated by agarose gel electrophoresis stained with GelRed^®^ (Biotium, Fremont, CA, USA). The mRNA integrity and concentration of samples used in the transcriptome analyses were evaluated with an Agilent 2100 Bioanalyzer (Agilent Technologies, Santa Clara, CA, USA).

### Transcriptomic Analysis

Whole transcriptomic expression profiles of hepatic RNA samples (n = 3) from each group were analyzed using a HiSeq X Ten system (Illumina, San Diego, CA, USA). STAR and edgeR were used to quantify transcriptomic data as previously described for differential gene expression within RNA samples (Robinson et al., 2010; Dobin et al., 2013). Changes in expression of genes affected by Thr deficiency were further confirmed by qPCR.

### Pathway Analysis With Gene Ontology and Kyoto Encyclopedia Genes and Genomes (KEGG)

Differentially expressed genes (|log_2_^Fold change^| > 0.585, P < 0.05) in liver were converted to their FASTA Protein Sequence, and gene enrichment was performed with the FASTA Protein Sequences by “Gene-list Enrichment” in Kobas 3.0 (http://kobas.cbi.pku.edu.cn/) (Wu et al., 2006; Xie et al., 2011). *Homo sapiens* was selected as the reference species, and hypergeometric test/Fisher’s exact test was applied as statistical method.

### qPCR

RNA samples were reverse transcribed to cDNA with the use of PrimerScript™ RT Master Mix (Code No. RR036A, Takara, Dalian, China) following the manufacturer’s instructions. qPCR analysis was performed using real-time PCR quantitative analysis in the fluorescence detection system (ABI Q7, Life Technologies, Shanghai, China) with Power Green Master Mix (Code No.4367659, Life Technologies, NY, USA). Primers used for amplification are listed in **Supplementary Table S2**. Glyceraldehyde 3-phosphate dehydrogenase (*GAPDH*) was used as a housekeeping reference gene to normalize the expression of the targeted genes (Vandesompele et al., 2002). Each specimen was measured independently in triplicate, and PCR amplification efficiency was close to 100%. Relative mRNA expression of target genes was calculated using the 2^-∆∆ct^ method as previously reported (Livak and Schmittgen, 2001).

### Statistical Analyses

Data were subjected to one-way ANOVA using general linear model procedure of SAS (version 9.2; SAS Institute Inc). Differences among means were tested by Duncan’s method. Data are expressed as mean and standard error of the mean (SEM). The level of statistical significance was set at *P* < 0.05.

## Results

### Growth Performance

Although the feed intake of ducks in the Pair-F group was assured to be similar to that of the Thr-D group by feed restriction, Thr intake, body weight, and weight gain in the Thr-D group were lower (P < 0.0001) than those in the Pair-F group (**Table 1**). Both Thr-D and Pair-F diets reduced (P < 0.0001) body weight and weight gain of ducks compared with that of the Thr-S group. The gain/feed ratio was lower in the Thr-D group than that in the Pair-F group (P < 0.03).

**Table 1:** Effects of threonine deficiency and feed restriction on growth performance of ducks from 1 to 21 days of age.

Items	Thr-D	Pair-F	Thr-S	Pooled SEM	*P*-value
Body weight (g)	984^c^	1029^b^	1275^a^	12.96	<0.0001
Weight gain (g/day)	44.3^c^	46.3^b^	58.0^a^	0.06	<0.0001
Feed intake (g/day)	75.6^b^	76.26^b^	96.9^a^	1.44	<0.0001
Gain : Feed (g/g)	0.59^b^	0.61^a^	0.60^ab^	0.01	0.0219
Thr intake (g/day)	0.40 ^c^	0.56 ^b^	0.71^a^	0.008	<0.0001

Thr-D, threonine deficiency; Thr-S, threonine sufficiency; Pair-F, pair-fed.

^a,b,c^Mean values with unlike superscript letters were significantly different (P < 0.05).

### Carcass Traits

Both Thr-D and Pair-F diets reduced the relative weights of breast muscle (P < 0.0001) and abdominal fat (P < 0.006) when compared to those of the Thr-S. These treatments had no effect (P > 0.05) on thigh muscle relative weight (**Table 2**). There were no differences in the relative weights of breast muscle and abdominal fat (P > 0.05) between Thr-D and Pair-F groups.

**Table 2:** Effects of threonine deficiency and feed restriction on carcass traits of ducks at 21 day of age.

Items	Thr-D	Pair-F	Thr-S	Pooled SEM	*P*-value
Breast muscle (% of live weight)	1.62^b^	1.54b	2.5^a^	0.063	<0.0001
Thigh muscle (% of live weight)	10.7	9.99	10.2	0.2741	0.2478
Abdominal fat (% of live weight)	0.76^b^	0.81b	0.9^a^	0.0248	0.0057

Thr-D, threonine deficiency; Thr-S, threonine sufficiency; Pair-F, pair-fed.

^a,b^Mean values with unlike superscript letters were significantly different (P < 0.05).

### Hepatic Lipid Accumulation

The Pair-F diet increased the relative weight of liver tissues compared with those of Thr-D and Thr-S (P < 0.0001), but there was no difference in the relative weight of liver between Thr-D and Thr-S (**Table 3**, P > 0.05). The Thr-D diet increased hepatic triglyceride concentration in Pekin ducks (P < 0.05) compared with those of the Thr-S and Pair-F groups. However, Thr-D and Pair-F treatments did not affect (P > 0.05) total lipids and cholesterol concentrations in liver.

**Table 3:** Effects of threonine deficiency and feed restriction on liver lipids of ducks at 21 day of age.

Items	Thr-D	Pair-F	Thr-S	Pooled SEM	*P*-value
Relative liver weight (%)*	3.64^b^	4.39^a^	3.29^b^	0.11	<0.0001
Total Lipid (% of fresh liver)	6.10	5.84	6.06	0.14	0.2502
Triglyceride (mg/g fresh liver)	6.66^a^	5.37^b^	5.48^b^	0.37	0.0432
Cholesterol (mg/g fresh liver)	1.66	1.35	1.70	0.11	0.0668

Thr-D, threonine deficiency; Thr-S, threonine sufficiency; Pair-F, pair-fed.

*Relative liver weight values were calculated as liver weight/liver body weight *×* 100%.

^a,b^Mean values with unlike superscript letters were significantly different (P < 0.05).

### Plasma Parameters

The Pair-F treatment elevated (P < 0.02) the glucose and HDLC concentrations and decreased LDLC (P < 0.001) levels compared with Thr-D and Thr-S diets (**Table 4**). Thr-D increased (P < 0.001) plasma HDLC concentration compared with the Thr-S diet, but its level was still lower than (P < 0.05) that in the Pair-F group. The Thr-D diet did not affect (P > 0.05) glucose and LDLC concentration compared with Thr-S group diet. Moreover, there were no significant differences in the plasma activities of ALT, AST, and ALP, or the plasma concentrations of CHO and TG between any groups.

**Table 4:** Effects of threonine deficiency and feed restriction on plasma parameters of ducks at 21 day of age.

Items	Thr-D	Pair-F	Thr-S	Pooled SEM	P-value
ALT (U/L)	40.5	37.9	41.6	1.54	0.2538
AST (U/L)	9.06	8.44	8.86	1.70	0.5064
ALP (U/L);	679	634	704	20.9	0.0894
GLU (mmol/L)	9.21^b^	9.69^a^	9.35^b^	0.11	0.0193
CHO (mg/dL)	7.13	7.14	6.91	0.15	0.4792
TG (mmol/L)	0.79	0.65	0.76	0.04	0.0586
HDLC (mmol/L)	3.90^b^	4.22^a^	3.64^c^	0.08	0.0009
LDLC (mmol/L)	2.04^a^	1.75^b^	2.21^a^	0.07	0.0008

Thr-D, threonine deficiency; Thr-S, threonine sufficiency; Pair-F, pair-fed; ALT, alanine transaminase; AST, aspartate transaminase; ALP, alkaline phosphatase; GLU, glucose; TG, total triglyceride; CHO, cholesterol; HDLC, high-density lipoprotein cholesterol; LDLC, low-density lipoprotein cholesterol.

^a,b,c^Mean values with unlike superscript letters were significantly different (P < 0.05).

### Liver Fatty acid composition

The Thr-D diet increased the hepatic contents of C6:0, C17:1, C18:3n6, C20:0, C20:1n9, and C22:2 and reduced the contents of C18:2n6t and C23:0 compared with the Thr-S diet (**Table 5**). However, there were no differences in total contents of fatty acid (TFA), saturated fatty acid (SFA), unsaturated fatty acid (USFA), and polyunsaturated fatty acid (PUFA) between Thr-D and Thr-S groups. The Pair-F diet increased the hepatic content of C14:0 and reduced the contents of C15:0, C17:0, C18:0, C20:0, C20:4n6, and C22:0 compared with those of Thr-D and Thr-S groups (P < 0.05). Moreover, the Pair-F group had lower total contents of fatty acid (TFA), saturated fatty acid (SFA), unsaturated fatty acid (USFA), and polyunsaturated fatty acid (PUFA) than those of Thr-D and Thr-S groups.

**Table 5:** Effects of threonine deficiency and feed restriction on hepatic fatty acid composition ducks at 21 day of age.

Fatty acid (mg/g)	Thr-D	Pair-F	Thr-S	Pooled SEM	*P*-value
C6:0	0.018^a^	0.012^b^	0.012^b^	0.001	0.001
C10:0	0.036	0.036	0.035	0.0002	0.247
C12:0	0.065	0.065	0.063	0.001	0.331
C14:0	0.210^b^	0.258^a^	0.208^b^	0.009	0.001
C14:1	0.039	0.039	0.035	0.001	0.073
C15:0	0.045^a^	0.043^b^	0.044^a^	0.0004	0.003
C15:1	0.048	0.047	0.048	0.001	0.615
C16:0	16.6^a^	14.4^b^	15.5^ab^	0.574	0.050
C16:1	0.556	0.690	0.624	0.041	0.101
C17:0	0.144^a^	0.114^b^	0.140^a^	0.004	< 0.0001
C17:1	0.043^a^	0.041^b^	0.041^b^	0.001	0.014
C18:0	16.0^a^	12.2^b^	15.0^a^	0.385	< 0.0001
C18:1n9c	21.1	17.0	17.0	1.59	0.144
C18:2n6t	0.067^b^	0.067^b^	0.070^a^	0.001	0.039
C18:2n6c	7.05	6.64	7.23	0.195	0.128
C18:3n6	0.089^a^	0.084^a^	0.067^b^	0.005	0.011
C18:3n3	0.105	0.087	0.110	0.008	0.172
C20:0	0.238^a^	0.211^c^	0.225^b^	0.004	0.0004
C20:1n9	0.297^a^	0.250^b^	0.260^b^	0.012	0.032
C20:2	0.936	0.802	0.873	0.038	0.075
C21:0	0.081	0.080	0.083	0.088	0.391
C20:3n6	1.20	1.28	1.26	0.039	0.317
C20:4n6	24.9^a^	21.0^b^	26.1^a^	0.792	0.001
C20:3n3	0.091	0.090	0.090	0.001	0.655
C20:5n3	0.142	0.179	0.186	0.014	0.079
C22:0	0.385^a^	0.349^b^	0.381^a^	0.006	0.001
C22:1	0.070	0.070	0.072	0.001	0.217
C22:2	0.134^a^	0.091^b^	0.092^b^	0.009	0.004
C23:0	0.213a^b^	0.202^b^	0.237^a^	0.007	0.011
C24:0	0.420	0.413	0.410	0.005	0.332
C22:6n3	0.958	1.02	1.04	0.050	0.473
C24:1	0.290	0.243	0.247	0.020	0.207
TFA	92.6^a^	78.0^b^	87.8^a^	2.80	0.007
SFA	34.6^a^	28.4^b^	32.4^a^	1.02	0.003
UFA	58.0^a^	49.7^b^	55.2^a^	1.78	0.015
MUFA	22.4	18.4	18.3	1.63	0.156
PUFA	35.7^a^	31.2^b^	37.1^a^	0.952	0.001

Thr-D, threonine deficiency; Thr-S, threonine sufficiency; Pair-F, pair-fed; TFA, total fatty acid; SFA, saturated fatty acid; UFA, unsaturated fatty acid; MUFA, monounsaturated fatty acid; PUFA, polyunsaturated fatty acid.

^a,b,c^Mean values with unlike superscript letters were significantly different (P < 0.05).

### Hepatic Transcriptome Analysis

**Figure 1:**
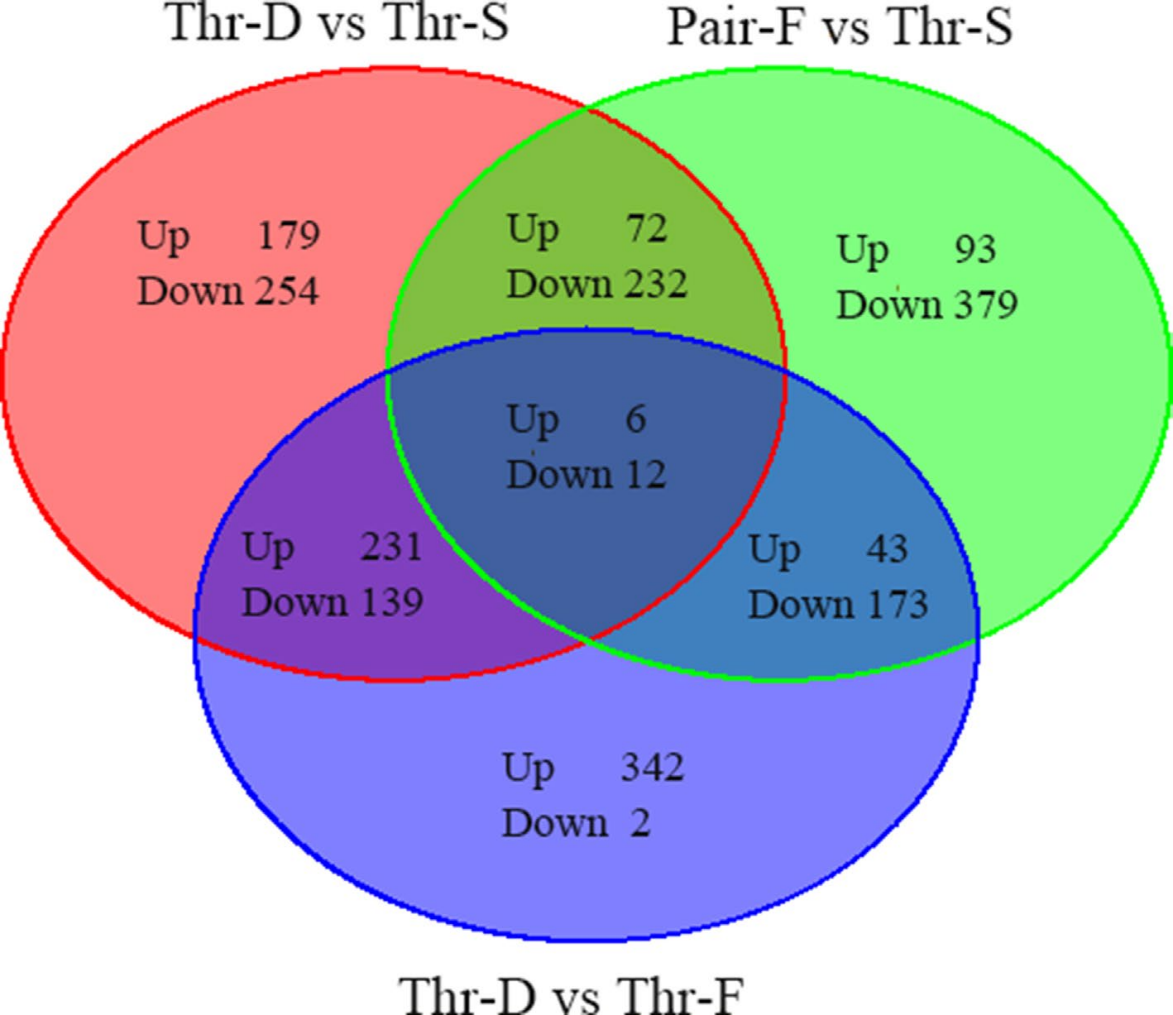
The total differentially expressed genes between groups (Thr-D vs Thr-S, Thr-S vs Pair-F, Pair-F vs Thr-S).

To screen differentially expressed genes in liver changed by Thr-D and Pair-F diets, a transcriptome analysis was performed in three individual duck livers from the different groups (Thr-D, Thr-S, and Pair-F). The differential genes were identified using *t*-test in EdgeR software (|Log_2_^Fold change^| > 0.585, *P* value < 0.05). Compared with the Thr-S group, Thr-D-fed animals had 1,125 differentially expressed genes: 488 were upregulated and 637 were downregulated (**Supplementary Table 3A**, **Figure 1**), and Pair-F-fed animals had 1,028 differentially expressed genes: 214 were upregulated and 814 were downregulated (**Supplementary Table 3B**, **Figure 1**). Compared with the Pair-F group, 948 genes were differentially expressed in the Thr-D group: 622 were upregulated and 326 were downregulated (**Supplementary Table 3C**, **Figure 1**).

### Transcriptome Data Validation by qPCR

**Figure 2:**
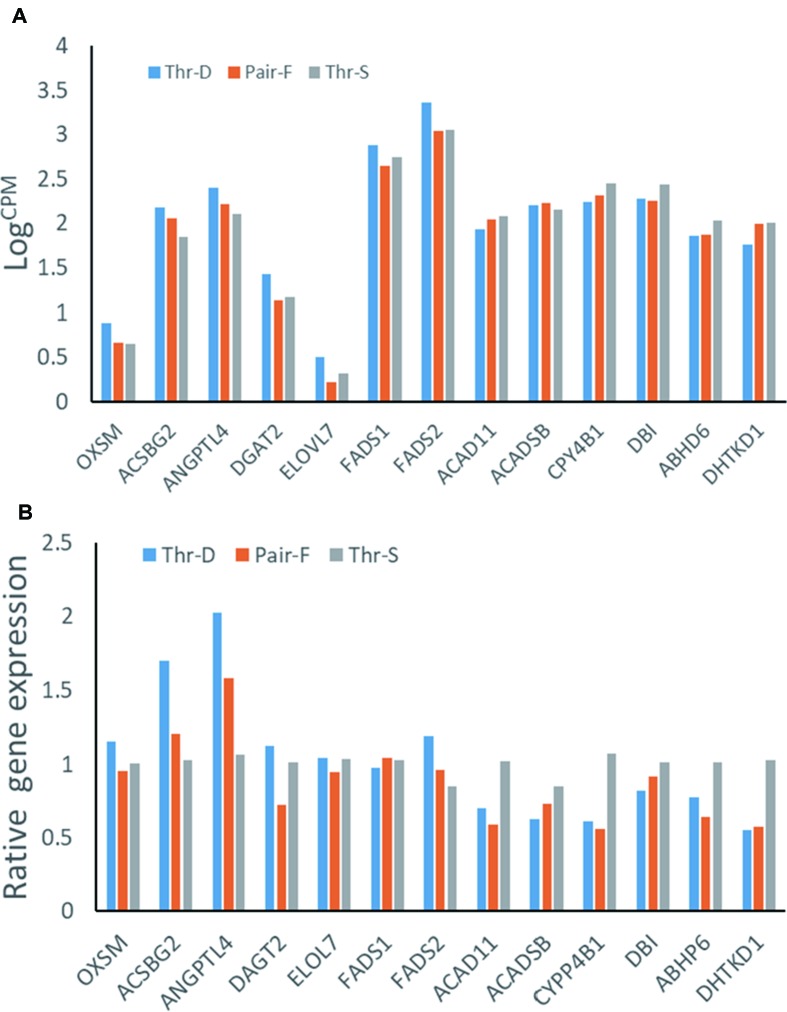
Gene expressions from transcriptome data **(A)** and qRT-PCR **(B)** of hepatic gene expressions of 21-day-old ducks from threonine defciency (Thr-D), threonine suffciency (Thr-S), and pair-fed (Pair-F). *FADS2*, fatty acid desaturase 2; *ACSBG2*, acyl-CoA synthetase bubblegum family member 2; *OXSM*, 3-oxoacyl-ACP synthase; *ELOVL7*, ELOVL fatty acid elongase 7;* FADS1*, atty acid desaturase 1; *DBI*, diazepam binding inhibitor, acyl-CoA binding protein; *DGAT2*, diacylglycerol O-acyltransferase 2; *ABHD6*, abhydrolase domain containing 6; *ACADSB*, acyl-CoA dehydrogenase, short/branched chain; *ACAD11*, acyl-CoA dehydrogenase family member 11; *CYP4B1*, cytochrome P450 family 4 subfamily B member 1; *DHTK1*, dehydrogenase E1 and transketolase domain containing 1; *ANGPTL4*, angiopoietin like 4. Data are means ± SEM (n = 6). ^a,b^Mean values with unlike superscript letters were significantly different (P < 0.05).

The hepatic gene expression patterns were verified using qPCR for 13 genes, which were selected to represent lipid metabolism pathways. The expression levels of 11 out of 13 tested genes were similar to those detected in the transcriptomic analysis (n = 6). The exceptions were *FADS1* and *ACADSB* (**Figure 2**).

### Gene Ontology and KEGG Pathway Analysis of Differential Genes

**Figure 3:**
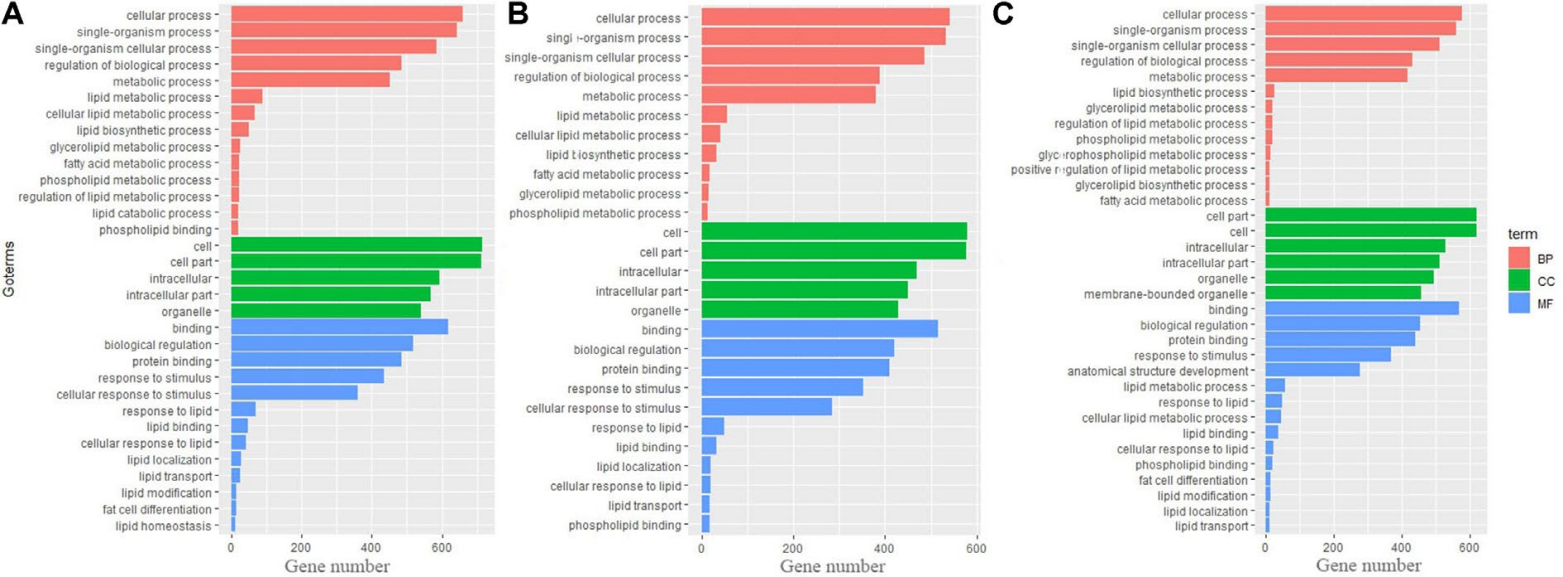
The GO terms involved in lipid metabolism enriched by differentially expressed genes (**A**, Thr-D vs Thr-S; **B**, Thr-D vs Pair-F; **C**, Pair-F vs Thr-D)

**Figure 4:**
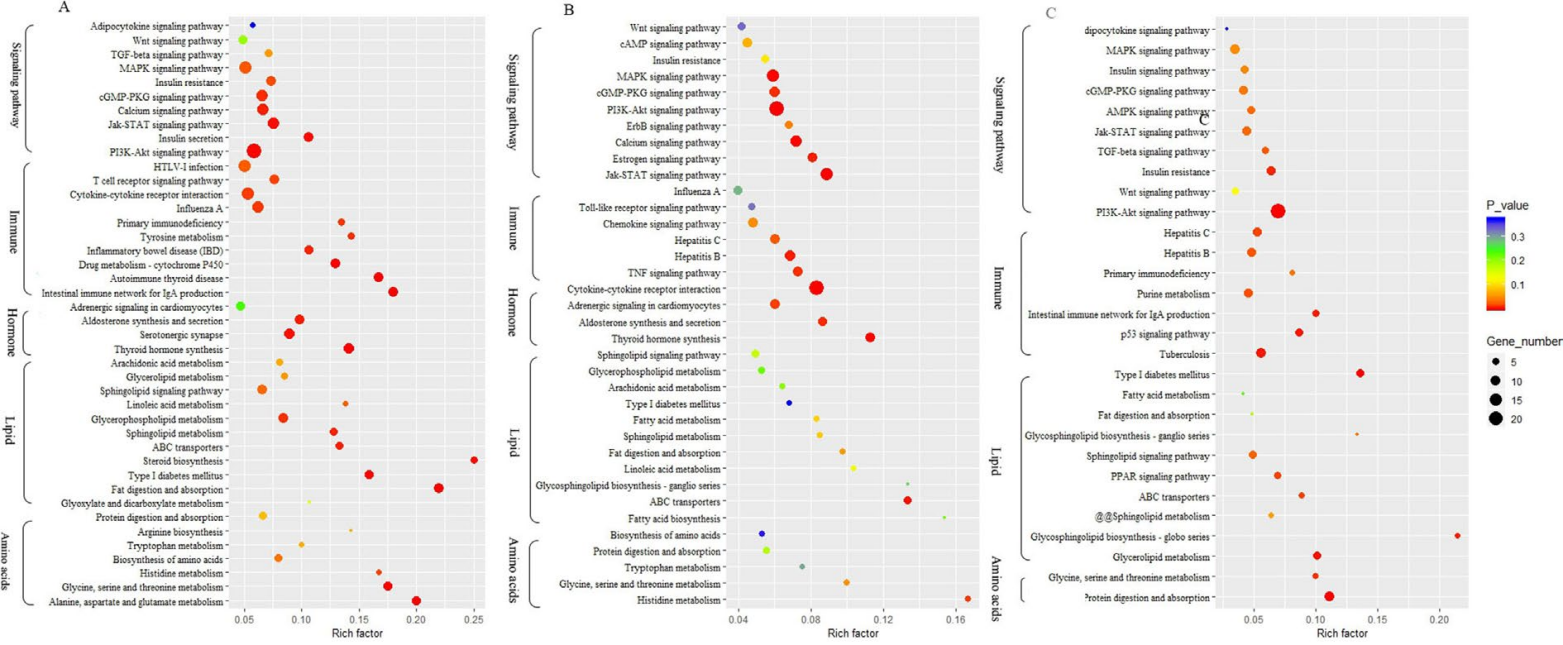
The KEGG pathway involved in lipid metabolism enriched by differentially expressed genes (**A**, Thr-D vs Thr-S; **B**, Thr-D vs Pair-F; **A**, Pair-F vs Thr-D)

To identify biological processes and KEGG pathways, the differentially expressed genes were decoded into their protein sequences. This resulted in 829, 682, and 733 protein sequences in Thr-D vs Thr-S, Thr-S vs Pair-F, and Pair-F vs Thr-S comparisons, respectively. An enrichment test was applied to search for significantly overrepresented GO terms (*P* value < 0.05) and KEGG pathways (*P* value < 0.05). The GO term enrichment showed that more genes related to lipid processes and molecular function were enriched in both Thr-D vs Thr-S and Thr-S vs Pair-F comparisons than in Pair-F vs Thr-S (**Figure 3**). Some KEGG pathways were also related to signal transduction, immunity, hormones, lipid, and amino acid metabolism (**Figure 4**).

## Discussion

Previous studies from our laboratory showed that dietary Thr deficiency increases hepatic triglyceride concentration and reduces the abdominal fat levels (Jiang et al., 2017; Jiang et al., 2019). In the present study, there were a reduction in growth and abdominal fat percentage and an increase in hepatic triglyceride concentration of ducks during dietary Thr deficiency (**Tables 1**, **2**, and **3**). Similar to those previous studies, we also observed a reduction in feed intake in the present study, which would affect physiological responses. Long-term (4–12 weeks) feed restriction reduces the concentration of ghrelin and increases the concentration of corticosterone in the plasma of turkeys (Vizcarra et al., 2018). In chickens, ghrelin activates the expression of corticotropin-releasing hormone and β-adrenergic receptor (Zendehdel and Hassanpour, 2014). In mammals, adrenaline can stimulate lipolysis by interacting with β-adrenergic receptor or inhibit lipolysis by interacting with α2-adrenergic receptor (Lafontan and Berlan, 1993; Robidoux et al., 2004). In addition, the reduction in feed intake reduces lipid deposition in cattle (Basarab et al., 2003). Therefore, the decreased feed intake itself observed in the present study might affect the hepatic lipid deposition in ducks.

Therefore, we assessed the role of feed restriction to distinguish its effects on hepatic triglyceride levels from those of Thr deficiency. By implementing feed restriction in the Pair-F group, we could assure that the feed intake of ducks in the Thr-D was similar to that of the Pair-F group. However, the growth performance of ducks in the Thr-D group was lower than that in the Pair-F group (**Table 1**), whereas ducks fed with the Thr-D diet increased their hepatic triglyceride concentration compared with those in the Pair-F group; Pair-F and Thr-S groups had similar hepatic triglyceride concentrations (**Table 4**). This indicates that low Thr intake directly increases hepatic triglyceride accumulation in ducks. These results differ from previous studies using broilers, in which feed restriction tends to reduce fat content in breast and thigh muscles (Wang et al., 2010) and reduce hepatic triglyceride content (Yang et al., 2010b). In lambs, feed restriction reduces total fatty acid, saturated fatty acid, and unsaturated fatty acid levels in the longissimus thoracis muscle (de Araújo et al., 2017). In the present study, the Pair-F group had increased hepatic content of C14:0 and reduced contents of C15:0, C17:0, C18:0, C20:0, C20:4n6, and C22:0, as well as reduced total fatty acid, saturated fatty acid, and unsaturated fatty acid contents, compared with Thr-D and Thr-S groups. The Thr-D group had increased hepatic fatty acid content (C6:0, C17:1, C18:3n6, C20:0, C20:1n9, and C22:2) compared with the Thr-S group. Ducks fed with the Pair-F diet had reduced hepatic fatty acid, whereas those fed with the Thr-D diet had increased hepatic triglyceride content. In addition, although there was a clear accumulation of triglycerides in the liver, its biological functions were not affected by Thr deficiency, as the plasmatic activities of AST, ALT, and ALP were unaffected by Thr-D and Pair-F diets. Nevertheless, Thr-D and Pair-F diets changed the concentration of GLU, HDLC, and LDLC in plasma, which indicates that Thr deficiency and feed restriction affect lipid metabolism.

In order to determine the underlying molecular changes caused by dietary Thr deficiency, we had previously assessed the expression of several genes involved in fatty acid synthesis and oxidation and triglyceride transport (Jiang et al., 2019). Unfortunately, the underlying mechanism of triglyceride accumulation caused by dietary Thr deficiency was still unclear after that study because of the large number of genes involved in the process. Therefore, here, we screened differentially expressed genes in response to dietary Thr deficiency using RNA sequencing techniques. It was observed that the lots of genes involved in amino acid, immune, energy, steroid, and lipid metabolism were differentially expressed.

### Amino Acid Biosynthesis

In the present study, 29 differentially expressed genes were associated with amino acid metabolism in the comparison of the Thr-D group against the Thr-S group, of which nine genes were upregulated and 20 genes were downregulated (**Supplementary Table 4A**). When compared with the Pair-F group, the Thr-D group presented nine upregulated genes and nine downregulated gene expression involved in amino acid metabolism (**Supplementary Table 4B**). The Pair-F diet affected the expression of 14 genes compared with the Thr-S diet, of which four genes were upregulated and 11 genes were downregulated (**Supplementary Table 4C**). Dynamic research about amino acid networks showed that Thr have positive effects on methionine, valine, isoleucine, leucine, asparagine, glutamic acid, tyrosine, cysteine, and a negative effect on serine pathways (Shikata et al., 2007; Shikata et al., 2010). A previous study from our laboratory showed that dietary deficiency reduces the plasmatic concentration of methionine, cysteine, lysine, arginine, isoleucine, valine, histidine, and phenylalanine and increases that of serine and alanine (Jiang et al., 2017). In the present study, the differentially expressed genes included those associated with alanine, histidine, aspartate, glutamate, glycine, serine, threonine, and tryptophan metabolism, and most of these genes were downregulated by the Thr-D diet (**Supplementary Table 4A**). In addition, genes involved in amino acid biosynthesis were downregulated by threonine deprivation (**Supplementary Table 5A**). These results indicate that amino acid biosynthesis decreased, whereas amino acid degradation increased in the liver of ducks fed with the Thr-D diet.

### Immunity

Threonine plays an important role in immune responses, as it affects the production of intestinal IgA in laying hens and pigs (Azzam et al., 2011; Zhang et al., 2017) and serum IgY in ducks (Zhang et al., 2014). In the present study, the enrichment analysis revealed that 57 genes were related to immune responses, including T cell and Toll-like receptor signaling in the comparison of the Thr-D against the Thr-S group (**Supplementary Table 5A**). The comparison between the Thr-D and the Pair-F groups resulted in 45 differentially expressed genes associated with immune responses (**Supplementary Table 5B**). Only 32 genes involved in immune responses were differentially expressed between Pair-F and Thr-S groups (**Supplementary Table 5C**). T cells play a key role in cell-mediated immunity and T cell receptor activation, and they enhance a number of signaling cascades that determine cell fate by regulating cytokine production, cell survival, proliferation, and differentiation (Burbach et al., 2007). We found that seven genes related to the T cell receptor signaling were downregulated, whereas only one was upregulated in the liver of animals fed with the Thr-D diet. This indicates that T cell immunity was repressed by threonine deprivation.

Toll-like receptors (TLRs) are key components of innate and adaptive immunities. They control host immune responses against pathogens through recognition of molecular patterns that are specific to microorganisms. In the present study, five genes associated with Toll-like receptor signaling were upregulated, and one gene was downregulated by threonine restriction. The nucleotide-sensing TLR3 is activated by double-stranded viral RNA, which is a sign of viral infection. Its activation leads to NF-κB induction, IRF3 nuclear translocation, cytokine secretion, and the inflammatory response *via* TRIF/TICAM1 (Oshiumi et al., 2003; Johnsen et al., 2006). Toll-like receptor 5 (TLR5) participates in the innate immune response to microbial agents (e.g., bacterial flagellin) by activating NF-κB and promoting cytokine secretion and the inflammatory response (Hayashi et al., 2001). In the present study, TLR3 and TLR5 were upregulated by the Thr-D diet compared with the Thr-S diet. Moreover, their downstream gene, MAP3K8, was also upregulated. In addition, compared with the Pair-F group, the Thr-D group upregulated five gene expression related to Toll-like receptor signaling. Genes affected by the Pair-F diet were not enriched in genes associated with TLR signaling and T cell receptor signaling. Taken together, our results show that the Thr-D diet regulates the TLR signaling by affecting the expression of genes related to both the activation of TLR signaling and its downstream effectors.

### Energy Metabolism

**Figure 5:**
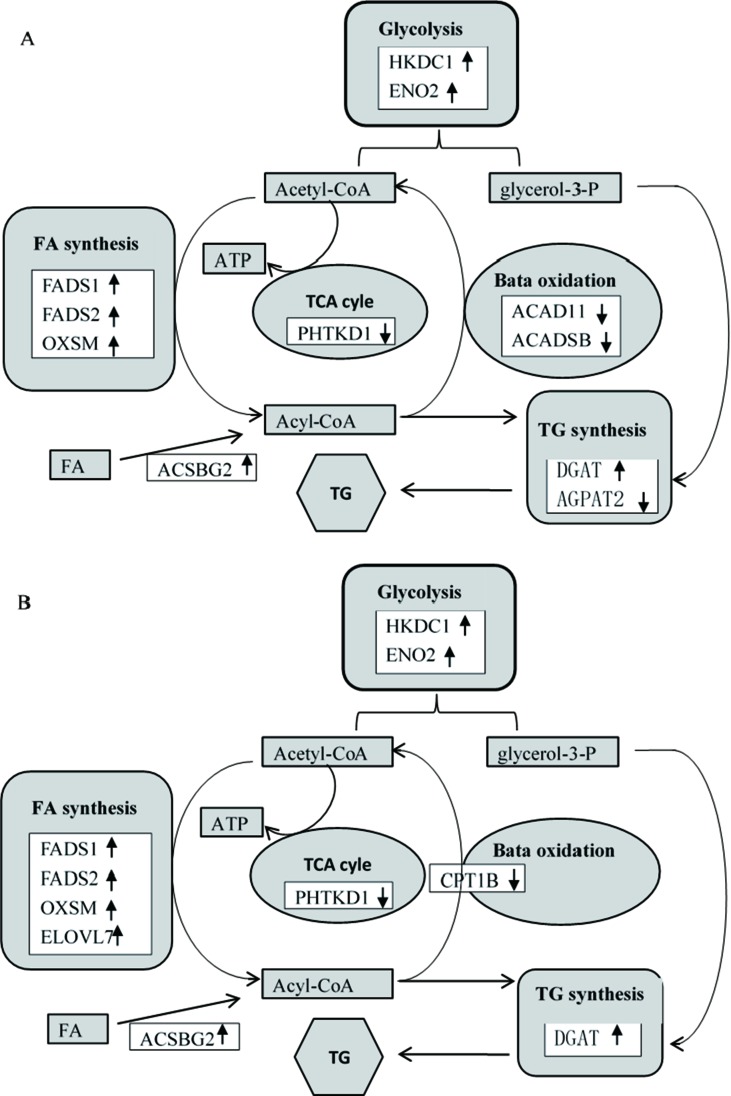
Differentially expressed genes (**A**, Thr-D vs Thr-S; **B**, Thr-D vs Pair-F) involved in glycolysis, fatty acid synthesis and beta oxidation, triglyceride synthesis, and tricarboxylic acid (TCA) cycle. The black up arrows at right indicate increased gene levels in response to threonine deficiency, and the black down arrows at right indicate reduced gene levels in response to threonine deficiency. *HKDC1*, hexokinase domain containing 1; *ENO2*, enolase 2; *FADS1*, fatty acid desaturase 1; *FADS2*, fatty acid desaturase 2; *OXSM*, 3-oxoacyl-ACP synthase; *ACADSB*, acyl-CoA dehydrogenase, short/branched chain; *ACAD11*, acyl-CoA dehydrogenase family member 11; *ACSBG2*, acyl-CoA synthetase bubblegum family member 2; AGPAT2, 1-acylglycerol-3-phosphate O-acyltransferase 2; *DGAT2*, diacylglycerol O-acyltransferase 2; *DHTKD1*, dehydrogenase E1 and transketolase domain containing 1; *ELOVL7*, ELOVL fatty acid elongase 7;* CPT1B*, carnitine palmitoyltransferase 1B.

Previous studies reported that dietary Thr deficiency reduces hepatic cell respiration in rats (Ross-Inta et al., 2009). In the present study, the Thr-D diet had increased expressions of enolase 2 (*ENO2*) and hexokinase domain containing 1 (*HKDC1*) and reduced the expression of dehydrogenase E1 and transketolase domain containing 1 (*DHTKD1*) and 3-hydroxymethyl-3-methylglutaryl-CoA lyase (*HMGCL*) genes compared with the that of Thr-S group (**Table 6**). One of those genes, *HKDC1*, encodes hexokinase, which phosphorylates hexoses (Wolf et al., 2011; Guo et al., 2015). Enolase, which catalyses the production of phosphoenolpyruvate, is encoded by the *ENO2* gene (Marangos and Schmechel, 1987). Glycolysis supplies glycerol 3-phoshate for triglyceride synthesis and acyl-CoA for fatty acid synthesis or tricarboxylic acid (TCA). In that regard, *DHTKD1* is a key gene for the TCA cycle, which codes the enzyme that converts 2-oxoglutarate to succinyl-CoA, generating ATP (Danhauser et al., 2012). A previous study showed that dietary Thr deficiency decreases liver mitochondrial coupling, leading to a reduction in ATP production (Ross-Inta et al., 2009). Another key metabolic pathway, ketogenesis, depends on the activity of HMGCL enzyme, which catalyses acetoacetate synthesis in the liver from 3-hydroxy-3-methylglutaryl-CoA (Tuinstra and Miziorko, 2003). Acetoacetate is transported to extrahepatic tissues (e.g., the brain) as energy source. In addition, the Thr-D diet induced the expressions of acyl-CoA synthetase short-chain family member 1 (*ACSS1*) and 3-hydroxybutyrate dehydrogenase 1 (*BDH1*) genes compared with that of the Pair-F group (**Table 6**). The protein encoded by *ACSS1* converts acetate to acetyl-CoA, used in the TCA cycle to produce energy and electron carriers, whereas BDH1 enzyme catalyses the interconversion of acetoacetate to 3-hydroxybutyrate (Churchill et al., 1992). 3-Hydroxybutyrate is absorbed by target tissues and converted back to acetoacetate by the same enzyme (Newman and Verdin, 2014). Acetoacetyl-CoA can be converted to two acetyl-CoA molecules, and then, they can be oxidized and produced ATP in TCA (Fukao et al., 2004). The Thr-D group had increased expressions of *ENO2* and *HKDC1* and reduced expression of *DHTKD1* compared with the Pair-P group (**Table 6**). However, there were no differences in the expression of these genes between Pair-F and Thr-S groups. Therefore, threonine deprivation increases glycolysis and affects ATP production and ketogenesis (**Figure 5**).

**Table 6:** Differentially expressed genes in liver involved in lipid metabolism on day 21 of ducks caused by threonine deficiency.

Genes	Annotation	Function	Log_2_^(Fold change)^*		
			Thr-D vs Thr-S	Thr-D vs Pair-F	Pair-F vs Thr-S
*ACOT12*	Acyl-coa thioesterase 12	Fatty acid synthesis	1.81	1.74	–
*FADS2*	Fatty acid desaturase 2	Fatty acid synthesis	0.90	1.05	–
*ACSBG2*	Acyl-coa synthetase bubblegum family member 2	Fatty acid synthesis	0.98	–	–
*OXSM*	3-oxoacyl-ACP synthase	Fatty acid synthesis	0.65	0.71	–
*ELOVL7*	ELOVL fatty acid elongase 7	Fatty acid synthesis	–	0.89	–
*FADS1*	Fatty acid desaturase 1	Fatty acid synthesis	–	0.73	–
*CPT1B*	Carnitine palmitoyltransferase 1B	Fatty acid degradation	–	-0.77	–
*ACADSB*	Acyl-coa dehydrogenase, short/branched chain	Fatty acid degradation	-0.79	–	–
*ACAD11*	Acyl-coa dehydrogenase family member 11	Fatty acid degradation	-0.59	–	–
*CYP4B1*	Cytochrome P450 family 4 subfamily B member 1	Fatty acid degradation	-0.82	–	–
*DBI*	Diazepam binding inhibitor, acyl-coa binding protein	Fatty acid transporter	-0.65	–	-0.70
*DGAT2*	Diacylglycerol O-acyltransferase 2	Triglyceride synthesis	0.71	0.94	–
*GPD1*	Glycerol-3-phosphate dehydrogenase 1	Triglyceride synthesis	1.16	1.32	–
*ABHD6*	Abhydrolase domain containing 6	Triglyceride degradation	-0.67	–	-0.62
*AGPAT2*	1-acylglycerol-3-phosphate O-acyltransferase 2	Phospholipid synthesis	-0.87	–	-0.65
*CHKA*	Choline kinase alpha	Phospholipid synthesis	2.43	2.08	–
*ETNK2*	Ethanolamine kinase 2	Phospholipid synthesis	-1.71	-1.33	–
*ENO2*	Enolase 2	Glycolysis	2.04	1.91	–
*HKDC1*	Hexokinase domain containing 1	Glycolysis	1.03	1.12	–
*DHTKD1*	Dehydrogenase E1 and transketolase domain containing 1	ATP production	-0.94	-0.80	–
*HMGCL*	3-hydroxymethyl-3-methylglutaryl-coa lyase	Ketogenesis	-1.63	–	–
*ACSS1*	Acyl-coa synthetase short-chain family member 1	Ketogenesis	–	0.60	–
*BDH1*	3-hydroxybutyrate dehydrogenase 1	Ketogenesis	–	0.90	–
*APOD*	Apolipoprotein D	Lipid transport	-0.69	–	-0.63
*ABCG1*	Cholesterol transport	Lipid transport	-1.23	–	-1.28
*SR-B1*	Scavenger receptor class B member 1	Lipid transport	-0.61	–	–
*IGFBP2*	insulin-like growth factor-binding protein 2	adipokine	-1.24	-1.05	–
*SOCS3*	suppressor of cytokine signaling 3	adipokine	1.53	2.46	-0.93
*ANGPTL4*	angiopoietin like 4	adipokine	0.90	–	–

Thr-D, threonine deficiency; Thr-S, threonine sufficiency; Pair-F, pair-fed.

*Log2^(Fold change)^ is expressed as the logarithmic fold change between the two of three groups. The positive Log2^(Fold change)^ indicates the upregulation genes, and the negative Log2^(Fold change)^ indicates downregulation genes.

### Lipid Metabolism

In the present study, 64, 31, and 30 genes that were related to lipid metabolism were differentially expressed between Thr-D and Thr-S, Thr-D and Pair-F, and Pair-F and Thr-S groups, respectively (**Supplementary Table 4A, B, C**). Among those, genes involved in fatty acid and triglyceride synthesis were upregulated, whereas genes related to fatty acid and triglyceride transport and degradation were downregulated by either Thr-D or Pair-F diets. The gene upregulated by the Thr-D diet included 3-oxoacyl-ACP synthase (*OXSM*), very long chain fatty acids protein 7 (*ELOVL7*), and long chain fatty acyl-CoA ligase (*ACSBG2*), which are involved in *de novo* synthesis of fatty acids (Zhang et al., 2005; Ohno et al., 2010; Naganuma et al., 2011). However, acyl-CoA dehydrogenase, short/branched chain (*ACADSB*), acyl-CoA dehydrogenase family member 11 (*ACAD11*), and cytochrome P450 (*CYP4B*) were downregulated by the Thr-D diet. These genes are involved in fatty acid β-oxidation and ω-oxidation (Vockley et al., 2000; He et al., 2011; Hsu et al., 2017; Roellecke et al., 2017; Scott, 2017). The Thr-D diet downregulated the expression of carnitine palmitoyltransferase 1B (*CPT1B*) compared with the Pair-F diet. The product of this gene is a protein that transports long chain acyl-CoA molecules from cytoplasm into mitochondria and thus controls a key regulatory step of β-oxidation (Kerner and Hoppel, 2000). In addition, Thr-D and Pair-F diets downregulated the expression of acyl-CoA binding protein (*DBI*), apolipoprotein D (*APOD*), and microsomal triglyceride transfer protein (*MTTP*). These genes participate in fatty acid and triglyceride transport (Rasmussen et al., 1994; Faergeman and Knudsen, 1997; Xie et al., 2006; Walsh et al., 2015). Based on the aforementioned mRNA results, it would be expected that the fatty acid content in Thr-D and Pair-F groups should be higher than that in the Thr-S group. However, we observed similar total fatty acid levels between Thr-D and Thr-S groups. In Thr-D and Pair-F groups, only the levels of C22:2, C20:1n9, C18:3n6, C18:2n6t, and C17:1 were higher than those in the liver of animals fed with the Thr-S diet. Moreover, hepatic triglyceride content in the Thr-D group was higher than that in the Thr-S group, but both groups had similar total lipids contents, which indicated that dietary Thr deficiency affected the hepatic lipid compositions. However, it was still unclear and need further experiments to prove it. Therefore, it was speculated that the synthesis of triglycerides is favored during dietary Thr deficiency. The expression of diacylglycerol O-acyltransferase 2 (*DGAT2*) was upregulated by the Thr-D diet compared with those by the other two dietary treatments. The protein coded by this gene binds diacylglycerol to long-chain fatty acid acyl-CoA in the final reaction of triglyceride biosynthesis (Agarwal et al., 2011; McFie et al., 2011; Qi et al., 2012). On the other hand, the Pair-F diet did not change the expression of *DGAT2* compared with the Thr-S group diet. This observation is consistent with that of similar levels of total lipids between the Thr-S and Pair-F groups.

### Steroid Biosynthesis

In the present study, the Thr-D diet upregulated the expression of 7-dehydrocholesterol r eductase (*DHCR7*), methylsterol monooxygenase 1 (*MSMO1*), NAD(P) dependent steroid dehydrogenase-like, lanosterol 14-alpha demethylase, squalene epoxidase (*SQLE*), and class I histocompatibility antigen compared with the Thr-S group diet. These genes are involved in steroid biosynthesis. To the best of our knowledge, there are no reports on the effects of dietary amino acid levels on the expression of genes related to steroid biosynthesis. However, feed restriction downregulates the expression of such genes in pigs and rats (Selman et al., 2006; Lkhagvadorj et al., 2009; Lkhagvadorj et al., 2010). In contrast, we found that the Pair-F diet did not change the expression of genes related to steroid biosynthesis compared with the Thr-S group diet. The discrepancy between the present study and previous studies might be due to species-specific characteristics.

### Effects of Thr on Adipocytokines

Adipocytokines play key roles in the regulation of lipid metabolism. In the present study, several adipocytokine factors involved in lipid metabolism were differentially expressed, including *SOCS3*, *IGFBP2*, and *ANGPTL4*. These genes have diverse functions, for example, *SOCS3* is a negative regulator of insulin signaling in skeletal muscle, adipose tissue, and the liver (Shi et al., 2004; Ueki et al., 2004; Yang et al., 2012), whereas it is also a negative regulator of leptin in the hypothalamus (Bjorbaek et al., 1999). The overexpression of *SOCS3* increases insulin and leptin resistance (Yang et al., 2012), which in turn increases hepatic lipid accumulation (Liu et al., 2014). In addition, *ANGPTL4* is expressed in the adipose tissue, liver, and placenta (Kersten et al., 2000; Kim et al., 2000; Yoon et al., 2000), where it regulates lipid metabolism primarily by inhibiting lipoprotein lipase activity (Ge et al., 2004; Köster et al., 2005) and promotes triglyceride uptake. The upregulation of *ANGPTL4* occurs in response to palmitic, oleic, arachidonic, and eicosapentaenoic acids (Gonzalez-Muniesa et al., 2011). Overexpression of *ANGPTL4* in mice increases the concentrations of triglycerides and cholesterol in plasma but does not increase body fat mass (Köster et al., 2005). Moreover, the overexpression of *ANGPL4* increases hepatic fat accumulation in mice (Xu et al., 2005). IGF binding protein 2 (*IGFBP2*) is expressed by several tissues, including the liver and white adipocytes (Gosteli-Peter et al., 1994), where it contributes to the prevention of diet-induced fat accumulation (Wheatcroft et al., 2007). Overexpression of *IGFBP2* reduces body fat accumulation in mice fed with a high-fat diet (Kammel et al., 2016) and decreases liver triglyceride deposition in ob/ob mice (Hedbacker et al., 2010). In the present study, the Thr-D diet downregulated *IGFBP2* and upregulated *ANGPTL4* and *SOCS3* in the liver of Pekin ducks, which agrees with the increased triglyceride deposition in the liver of these animals.

### Effects of Thr on Pathway Related to Lipid Metabolism

In addition, we found that several cellular regulatory pathways involved in lipid metabolism were differentially expressed in Thr-D and Pair-F groups, such as PI3K-Akt, Jak-STAT, cGMP-PKG, and Wnt signaling pathway. Apoptosis-related pathways, such as PI3K/AKT-TOR, cAMP/PKA/CREB, and LKB1/AMPK-FOXO, regulate methionine-induced changes in hepatic lipid metabolism in fish and mammals (Gao et al., 2018). Signal transduction in the Jak-STAT pathway has been demonstrated to regulate lipid metabolism in mice (Xu et al., 2013; Shi et al., 2014). For instance, di-(2-ethylhexyl) phthalate reduces lipid hydrolysis and promotes triglyceride accumulation by regulating the activation state of the Jak-STAT pathway in the liver and adipose tissue in rats (Jia et al., 2016) and magnesium-induced reduction in hepatic lipid deposition in yellow catfish (Wei et al., 2017). In the present study, the Thr-D diet downregulated the expression of signal transducer and activator of transcription 4 (*STAT4*) and upregulated the expression of suppressor of cytokine signaling 3 (*SOCS3*), whereas the Pair-F diet did not change the expression of *STAT4* but downregulated the expression of *SOCS3* compared with the Thr-S group diet. Cytokines activate the JAK-STAT signaling pathway by binding to its receptor, leading to the expression of *SOCS* (Krebs and Hilton, 2000). The upregulated SOCS protein acts through a feedback mechanism to inhibit the JAK-STAT signaling pathway in the cytosol (Miao et al., 2006). Therefore, in Pekin ducks, the high expression of *SOCS3* might inhibit that of *STAT4* in the liver of animals fed with the Thr-D diet.

Activation of PI3K/AKT might lead to the expression of Fas cell surface death receptor and Sterol regulatory element-binding proteins (Jeon and Osborne, 2012; Liu et al., 2016). On the other hand, inhibition of PI3K/AKT impairs the suppression of very-low-density lipoprotein assembly and insulin secretion by regulating the secretion and degradation of apolipoprotein B and the gene expression of hepatic *MTTP* (Sidiropoulos et al., 2007). Moreover, inhibition of PI3K/AKT/mTOR pathway reduces hepatic lipid accumulation by increasing fatty acid oxidation and VLDL assembly and secretion in geese (Liu et al., 2016). In the present study, the Thr-D diet upregulated eight genes and downregulated 12 genes involved in the PI3K-AKT signaling pathway, as well as downregulated the expression of *MTTP*, compared with the Thr-S group diet. Feed restriction in the Pair-F group upregulated 15 genes and downregulated 59 genes in PI3K-AKT signaling pathway, when compared with that in the Thr-S group. Thus, it is clear that both Thr-D and Pair-F diets changed the expression of genes that compose the PI3K-AKT signaling pathway, but there were differences between Thr-D and Pair-F groups.

In summary, both Thr-D and Pair-F diets affected hepatic lipid metabolism in Pekin ducks. However, the molecular mechanisms underlying lipid metabolism changes differed between Thr-D and Pair-F groups. Animals in the Pair-F group had reduced hepatic individual fatty acid contents, whereas ducks in the Thr-D group had a similar fatty acid profile when compared with those in the Thr-S group. However, the Thr-D diet increased the accumulation of hepatic triglycerides, and the Pair-F diet had no effect on hepatic triglyceride accumulation. Transcriptome analysis showed that the Thr-D diet upregulated the expression of genes related to fatty acid and triglyceride synthesis and downregulated genes related to fatty acid oxidation and triglyceride transport. This indicates that threonine deprivation promoted the entrance of fatty acids into the triglyceride pathway. Both Thr-D and Pair-F diets induced regulatory changes involved in lipid metabolism that might be controlled by PI3K-AKT, Jak-STAT, and adipocytokine signaling pathways. The role of these signaling pathways in the regulation of lipid metabolism during dietary threonine deprivation warrants further research.

## Data Availability

The datasets generated for this study can be found in NCBI. The number of BioProject is PRJNA530027, and the data can be visited at https://www.ncbi.nlm.nih.gov/bioproject/PRJNA530027.

## Ethics Statement

The study was approved by the Animal Management Committee (in charge of animal welfare issue) of the Institute of Animal Science, Chinese Academy of Agricultural Sciences (IAS20160322, IAS-CAAS, Beijing, China) and performed in accordance with the guidelines. Ethical approval on animal survival was given by the animal ethics committee of IAS-CAAS.

## Author Contributions

SH conceived and coordinated the study. YJ performed the study, was involved in all aspects of analysis, and drafted the manuscript. MX and SH were involved in experimental design. MX performed data analysis. JT, WF, and JX performed the sample analysis. GC participated in editing the manuscript. SH had primary responsibility for the final content. All authors read and approved the final version of the manuscript.

## Funding

This work was supported by the earmarked fund for China Agriculture Research System (CARS-42) and the science and technology innovation project of Chinese Academy of Agricultural Sciences (CXGC-IAS-09).

## Conflict of Interest Statement

The authors declare that the research was conducted in the absence of any commercial or financial relationships that could be construed as a potential conflict of interest.
