# Supplementary material for: Transcriptome Analysis Reveals Differential Expression of Genes Regulating Hepatic Triglyceride Metabolism in Pekin Ducks During Dietary Threonine Deficiency
Source: Front Genet. 2019 Aug 2;10:710. doi: 10.3389/fgene.2019.00710 (PMC6688585; doi:10.3389/fgene.2019.00710)
Supplement: Supplementary file 1 [file Table_1.docx]

Supplemental Table S1. Composition of basal diets for Pekin ducks from 1 to 21 d of age as-fed basis

| Ingredients % |  | Composition % |  |
| --- | --- | --- | --- |
| Corn | 26.27 | Metabolizable energy (Kcal/kg) ‡ | 2951 |
| Corn gluten meal | 2.00 | Crude protein§ | 19.91 |
| Soybean oil | 1.00 | Lysine§ | 1.09 |
| Wheat | 49.00 | Tryptophan§ | 0.23 |
| Peanut meal | 16.50 | Methionine § | 0.49 |
| Dicalcium phosphate | 1.90 | Methionine + Cystine§ | 0.82 |
| Limestone | 1.13 | Arginine§ | 1.44 |
| Salt | 0.30 | Threonine§ | 0.53 |
| Methionine | 0.22 | Valine§ | 0.91 |
| tryptophan | 0.06 | Isoleucine§ | 0.70 |
| Lysine | 0.64 | Calcium‡ | 0.90 |
| Valine | 0.12 | Total phosphorus‡ | 0.68 |
| Isoleucine | 0.06 | Nonphytate phosphorus ‡ | 0.45 |
| Premix† | 0.50 |  |  |
| Corn starch +threonine* | 0.30 |  |  |
| Total | 100 |  |  |

†Supplied per kilogram of total diet: Cu (CuSO_4_•5H_2_O), 8 mg; Fe (FeSO_4_•7H_2_O), 60 mg; Zn (ZnO), 60 mg; Mn (MnSO_4_•H_2_O), 100 mg; Se (NaSeO_3_), 0.3 mg; I (KI), 0.4 mg; choline chloride, 1,000 mg; vitamin A (retinyl acetate), 4,000 IU; vitamin D_3_ (Cholcalciferol), 2,000 IU; vitamin E (DL-α-tocopheryl acetate), 20 IU; vitamin K_3_ (menadione sodium bisulphate), 2 mg; thiamin (thiamin mononitrate), 2 mg; riboflavin, 10 mg; pyridoxine hydrochloride, 4 mg; cobalamin, 0.02 mg; calcium-D-pantothenate, 20 mg; nicotinic acid, 50 mg; folic acid, 1 mg; and biotin, 0.15 mg.

*crystalline threonine supplements added in place of equivalent weights of cornstarch.

‡ These values are as formulated.

§These values determined by analysis based on triplicate determinations.

Supplemental Table S2. Primer sequences for real-time PCR amplification

| Genes | Gene Bank ID | Product, bp | Primer sequences |
| --- | --- | --- | --- |
| *GAPDH* | XM_0050167 | 104 | F: 5'-AGATGCTGGTGCTGAATACG-3' |
|  |  |  | R: 5'-CGGAGATGATGACACGCTTA-3' |
| *FADS2* | XM_013103963.1 | 81 | F: 5'-TGCAACATCGAGCAGTCCTT-3' |
|  |  |  | R: 5'-TGTTGGAAACAGGTGGTGCT-3' |
| *ACSBG2* | XM_013108256.1 | 238 | F: 5'-TTGCTGCACAGATGACGGAT-3' |
|  |  |  | R: 5'-TGTAACCCGACTTCCTTGGC-3' |
| *OXSM* | XM_005029252.2 | 141 | F: 5'-CCGAATCACGCTGTGTCAAC-3' |
|  |  |  | R: 5'-TCCAGCCAAGGACAAAGGAC-3' |
| *ELOVL7* | XM_005008915.2 | 174 | F: 5'-AGCACTGGTTACCTTGCCTC-3' |
|  |  |  | R: 5'-GCGTGTGTGCCCTTAACAAT-3' |
| *FADS1* | XM_005024262.2 | 181 | F: 5'-TTCCGTGAACTCCGTGTAGC-3' |
|  |  |  | R: 5'-CCTGGACAGTGCCTAGAAGC-3' |
| *DBI* | XM_005020573.2 | 278 | F: 5'-AGGCAAAGCAAAGTGGGATG-3' |
|  |  |  | R: 5'-AGCACGTCAGTACATTAGGCA-3' |
| *DGAT2* | XM_005024101.3 | 280 | F: 5'-CTATTTTGGGGAGGGCAGCA-3' |
|  |  |  | R: 5'-CAGCCACCAAAGTATCCGGT-3' |
| *ABHD6* | XM_021276856.1 | 217 | F: 5'-GGGAACGTCGCTGGAGTTTA-3' |
|  |  |  | R: 5'-TGAAGCGAACGTAGGAGCAG-3' |
| *ACADSB* | XM_021274901.1 | 99 | F: 5'-CGAGGCTGCTGACCTACAAT-3' |
|  |  |  | R: 5'-TGGCAACCTCTGCAGCATAA-3' |
| *ACAD11* | XM_021274262.1 | 258 | F: 5'-CAGTGCTGGACTGGGAACTT-3' |
|  |  |  | R: 5'-CCTGCGATATCCCTGCCATT-3' |
| *CYP4B1* | XM_013106764.1 | 128 | F: 5'-GTGCCAAGGACGAGAATGGA-3' |
|  |  |  | R: 5'-CAGTGCCAGGCAGTACAAGA-3' |
| *DHTK1* | XM_013097534.2 | 282 | F: 5'-AGGAACCTTTAGCCAGCGAC-3' |
|  |  |  | R: 5'-CCACTCTGCAGGAGCCATTT-3' |
| *ANGPTL4* | XM_005027210.3 | 290 | F: 5'-CTCGTCCAGGAGGGAAGGTA-3' |
|  |  |  | R: 5'-TACTTCCCGTTGAGGTTGGC-3' |

*FADS2*= fatty acid desaturase 2; *ACSBG2=* acyl-CoA synthetase bubblegum family member 2; *OXSM* = 3-oxoacyl-ACP synthase; *ELOVL7*= ELOVL fatty acid elongase 7; *FADS1*=atty acid desaturase 1; *DBI* = diazepam binding inhibitor, acyl-CoA binding protein; *DGAT2*= diacylglycerol O-acyltransferase 2; *ABHD6*= abhydrolase domain containing 6; *ACADSB* = acyl-CoA dehydrogenase, short/branched chain; *ACAD11*= acyl-CoA dehydrogenase family member 11; *CYP4B1*= cytochrome P450 family 4 subfamily B member 1; *DHTK1*= dehydrogenase E1 and transketolase domain containing 1; *ANGPTL4*= angiopoietin like 4.
