# Supplementary material for: Transcriptome Analysis Reveals Differential Expression of Genes Regulating Hepatic Triglyceride Metabolism in Pekin Ducks During Dietary Threonine Deficiency
Source: Front Genet. 2019 Aug 2;10:710. doi: 10.3389/fgene.2019.00710 (PMC6688585; doi:10.3389/fgene.2019.00710)
Supplement: Supplementary file 2 [file Table_2.docx]

Supplemental Table S2. Primer sequences for real-time PCR amplification

| Genes | Gene Bank ID | Product, bp | Primer sequences |
| --- | --- | --- | --- |
| *GAPDH* | XM_0050167 | 104 | F: 5'-AGATGCTGGTGCTGAATACG-3' |
|  |  |  | R: 5'-CGGAGATGATGACACGCTTA-3' |
| *FADS2* | XM_013103963.1 | 81 | F: 5'-TGCAACATCGAGCAGTCCTT-3' |
|  |  |  | R: 5'-TGTTGGAAACAGGTGGTGCT-3' |
| *ACSBG2* | XM_013108256.1 | 238 | F: 5'-TTGCTGCACAGATGACGGAT-3' |
|  |  |  | R: 5'-TGTAACCCGACTTCCTTGGC-3' |
| *OXSM* | XM_005029252.2 | 141 | F: 5'-CCGAATCACGCTGTGTCAAC-3' |
|  |  |  | R: 5'-TCCAGCCAAGGACAAAGGAC-3' |
| *ELOVL7* | XM_005008915.2 | 174 | F: 5'-AGCACTGGTTACCTTGCCTC-3' |
|  |  |  | R: 5'-GCGTGTGTGCCCTTAACAAT-3' |
| *FADS1* | XM_005024262.2 | 181 | F: 5'-TTCCGTGAACTCCGTGTAGC-3' |
|  |  |  | R: 5'-CCTGGACAGTGCCTAGAAGC-3' |
| *DBI* | XM_005020573.2 | 278 | F: 5'-AGGCAAAGCAAAGTGGGATG-3' |
|  |  |  | R: 5'-AGCACGTCAGTACATTAGGCA-3' |
| *DGAT2* | XM_005024101.3 | 280 | F: 5'-CTATTTTGGGGAGGGCAGCA-3' |
|  |  |  | R: 5'-CAGCCACCAAAGTATCCGGT-3' |
| *ABHD6* | XM_021276856.1 | 217 | F: 5'-GGGAACGTCGCTGGAGTTTA-3' |
|  |  |  | R: 5'-TGAAGCGAACGTAGGAGCAG-3' |
| *ACADSB* | XM_021274901.1 | 99 | F: 5'-CGAGGCTGCTGACCTACAAT-3' |
|  |  |  | R: 5'-TGGCAACCTCTGCAGCATAA-3' |
| *ACAD11* | XM_021274262.1 | 258 | F: 5'-CAGTGCTGGACTGGGAACTT-3' |
|  |  |  | R: 5'-CCTGCGATATCCCTGCCATT-3' |
| *CYP4B1* | XM_013106764.1 | 128 | F: 5'-GTGCCAAGGACGAGAATGGA-3' |
|  |  |  | R: 5'-CAGTGCCAGGCAGTACAAGA-3' |
| *DHTK1* | XM_013097534.2 | 282 | F: 5'-AGGAACCTTTAGCCAGCGAC-3' |
|  |  |  | R: 5'-CCACTCTGCAGGAGCCATTT-3' |
| *ANGPTL4* | XM_005027210.3 | 290 | F: 5'-CTCGTCCAGGAGGGAAGGTA-3' |
|  |  |  | R: 5'-TACTTCCCGTTGAGGTTGGC-3' |

*FADS2*= fatty acid desaturase 2; *ACSBG2=* acyl-CoA synthetase bubblegum family member 2; *OXSM* = 3-oxoacyl-ACP synthase; *ELOVL7*= ELOVL fatty acid elongase 7; *FADS1*=atty acid desaturase 1; *DBI* = diazepam binding inhibitor, acyl-CoA binding protein; *DGAT2*= diacylglycerol O-acyltransferase 2; *ABHD6*= abhydrolase domain containing 6; *ACADSB* = acyl-CoA dehydrogenase, short/branched chain; *ACAD11*= acyl-CoA dehydrogenase family member 11; *CYP4B1*= cytochrome P450 family 4 subfamily B member 1; *DHTK1*= dehydrogenase E1 and transketolase domain containing 1; *ANGPTL4*= angiopoietin like 4.
